# Editorial

**Published:** 1848-07

**Authors:** 


We publish in this No. of the Register the remarks of the Editor of the Dental Recorder—drawn out by a review of the American Society's proceedings, by my brother E. Taylor—now and for a year past associated with me in practice.

Wishing to do ample justice to every member of the Profession and particularly the Editor of the Recorder, for whom we entertain the kindest feelings—we insert his remarks entire. His oversight in taking my brother for the Cincinnati Editor, renders it necessary that we should say a few words which may fully explain our position to the American Society of Dental Surgeons. To do this aright we must go back some three years, and by reference to the September No. Vol. 5, No. 1, of American Journal and Library of Dental Science. It will be observed that we attended the 6th annual meeting of that society; that when the subject of Amalgams came up, a committee was appointed to visit all the members of the Society then in the city, and ascertain from each whether he had "used any amalgam in the course of his own practice as a dental surgeon, or approves of its use." We were one of the committee, but owing to the fact, that we were a stranger in the city, and to most of the dentists, the other members of the committee kindly performed the duty assigned us. After this another committee of five was appointed to report "some plan of action for the Society to take in this matter," and we were of that committee. For our report we refer to the published proceedings in the Journal. The plan agreed upon by the committee at that time appeared to be the quickest and most effectual method of getting rid of this matter, and there appeared to be such a desire by the members of the Society to have the question finally disposed of, that we the more readily assented to the proceeding. We shall always admire the noble self-sacrificing spirit manifested by our friend, Dr. Baker, who, when pressed upon this question, replied, that although he could not agree with different members of the Society as it regards the proper use of this article; yet if they thought its use prejudicial to the interest of the Profession he would relinquish it. We merely give, so far as we can recollect, the substance of his remarks. We thought at the time, and still think it as far as we could ask any man to go. But there are other considerations which induced our consent to the report submitted. The Mississippi Valley Association of Dental Surgeons, at its formation, took ground against the use of this filling; and the practice of amalgam filling in the West was almost entirely in the hands of a set of itinerating quacks, who knew not how to use aright this or any other substance for filling teeth—and we were aware that some such, West of the Allegheny had by hook or crook, worked themselves into the American Society, and who should have been expelled, or rather should never have been admitted. It was to rid the Society of these *blotches*, who not only disgraced the Society but the Profession at large, that we consented to the plan recommended by that committee. True, they might have been arraigned for mal-practice and expelled. This would have been perhaps the constitutional method. Had we known so many of our eastern brethren were so fully committed to the use of this "nasty stuff," we should much prefer having had the question thus disposed of.

Thus far, it will be observed, we go with the society. At this stage of the proceedings we left, and we believe our name is recorded on the first protest. We have always supposed this *all sufficient*, and never once thought that the members of the Society then present, and who had signed this, would ever be asked to sign another. We have, therefore, neglected or refused to sign one or two others, which have been sent us; at the time supposing that they were sent inadvertantly, or perhaps by the Secretary, merely to let us know the thing was being done to the members who were not in attendance at the meeting, according to the expressed will of the Society. We still think it was entirely unnecessary to have sent the protest to any who had signed the instrument at that meeting: and the mere fact that the form was somewhat changed thereafter, does not alter the case, for the first asks all, embraces all that could be desired. This, then, is the ground on which we stand. We cannot consent to give any stronger protest against the use of amalgam fillings, than we have; and if we are hereafter expelled, it will be, not because we do not discard this substance in our practice—not because we refused not to give our protest at the time it was proper to give it, but because we have since refused to reiterate and reiterate the same and similar protests. If we cannot be believed on the first statement of a fact, we ask not to be believed by any farther assertion we can make.

The Editor of the Dental Recorder, therefore, mistakes not only the man, but even position in relation to this very great, and at the east *absorbing* question. We are truly glad that with *us;* there is such unanmity in discarding this substance in our practice; and are free to confesr, that, as we grow older in practice, we become more extravagant in the use of gold; and when we have succeeded in preserving a tooth very much decayed, with this article as a filling, we feel that we have done our patient a far greater service, than if we had used anything else. The question, to what extent old roots and dead shells of teeth may be made serviceable by the use of amalgam, we have not decided, except, indeed, to this extent, which is, that we prefer taking them out entirely.

The note by our brother, which is appended to tbe article from the Dental Recorder, renders it necessary for us to say more on this subject.—CIN. ED.

